# Prognostic Factors and Nomogram for Malignant Brainstem Ependymoma: A Population‐Based Retrospective Surveillance, Epidemiology, and End Results Database Analysis

**DOI:** 10.1002/cam4.70564

**Published:** 2025-01-17

**Authors:** Xiaoyu Ji, Siyuan Yang, Dejing Cheng, Wenbo Zhao, Xuebo Sun, Fang Su

**Affiliations:** ^1^ Department of Neurosurgery The First Affiliated Hospital of Soochow University Suzhou China; ^2^ The Fourth Affiliated Hospital of Soochow University Suzhou China; ^3^ Department of Neurosurgery Second Hospital of Shanxi Medical University Taiyuan Shanxi Province China

**Keywords:** brainstem ependymoma, mortality, prognosis, SEER, survival

## Abstract

**Purpose:**

This study aimed to identify prognostic factors and develop a nomogram for survival in patients with brainstem ependymoma.

**Methods:**

Data of 652 patients diagnosed with brainstem ependymoma extracted from the Surveillance, Epidemiology, and End Results (SEER) registry from 2000 to 2020 were analyzed. Univariate and multivariable Cox regression analyses were performed to examine factors influencing overall survival (OS). Receiver operating characteristic curve (ROC) and calibration curves were used to verify the nomogram. The Kaplan–Meier method was used to analyze OS based on treatment methods stratification or different age patterns.

**Results:**

Six independent prognostic factors of patients with brainstem ependymoma were identified, including age, race, marital status, radiation, gross total resection (GTR), and histology. A comprehensive nomogram model was developed utilizing these predictors identified through multivariable Cox regression analysis. Furthermore, we found that patients with GTR have improved overall survival than patient with no surgery and biopsy only or with partial resection (GTR vs. no: *p* = 0.0004, GTR vs. partial resection: *p* = 0.022). Patients with radiation have improved overall survival than patient without radiation (*p* = 0.00013). Patients with GTR combined radiation therapy have improved overall survival than patient without or with GTR or radiation therapy only (*p* < 0.0001). Different treatment methods have no significant difference in the overall survival probability of the elderly group.

**Conclusions:**

Individuals who are Black and anaplastic ependymomas were negative risk factors for brainstem ependymoma associated with an increased risk of mortality. Patients aged < 50 years with GTR and radiation always had better survival.

**Abbreviations**
GTR: gross total resection
K‐M: Kaplan–Meier
OS: overall survival
PFS: progression‐free survival
ROC: operating characteristic curve
SEER: Surveillance, Epidemiology, and End Results

## 1 Introduction

Ependymomas are central nervous system tumors that primarily develop from ependymal cells or the central canal of the spinal cord. These tumors exhibit distinct patterns of occurrence based on age: in children, ependymomas are most frequently located in the posterior fossa, while in adults, they are more commonly found in the spinal cord and supratentorial regions [1]. Typically, ependymomas manifest as solid masses arising from the ventricular lining of the fourth ventricle. However, it is relatively uncommon for these tumors to originate from the brainstem, a region of critical importance for basic life functions [2].

Brainstem ependymoma is a rare and highly aggressive tumor of the central nervous system [3]. Due to the unique anatomical location and function of the brainstem, traditional treatments for tumors in this region are technically extremely demanding and fraught with risk. Surgical interventions, radiotherapy, and chemotherapy in this area are complicated by the need to preserve critical neurological functions, leading to a higher risk of complications and limited treatment success. As a result, patients diagnosed with brainstem ependymoma often have a poor prognosis with limited survival benefits and a complex clinical course [4, 5].

A nomogram is a graphical representation that scores various risk factors to predict the prognosis of tumors, and it has become a crucial tool in clinical practice. This study utilized the Surveillance, Epidemiology, and End Results (SEER) program data from the National Cancer Institute. The SEER program provides comprehensive cancer statistics covering approximately 35% of the US population based on the 2000 census, offering a rich resource for epidemiological research and analysis [6]. Furthermore, there are no study researching the prognosis of patients with brainstem ependymoma via the SEER database. In this study, we leveraged the extensive and detailed data from the SEER database to elucidate the prognostic factors associated with the overall survival of patients with brainstem ependymoma. By identifying these factors, we subsequently constructed a predictive nomogram and also analyze patients' overall survival (OS) based on treatment methods stratification or different age patterns.

## 2 Methods

### 2.1 Study Population

The study utilized accessible clinical data from SEER*Stat software. Given that the SEER database offers publicly accessible research data, our institutional review board has exempted the SEER database from review and has waived the requirement for informed consent. The data with malignant brainstem tumors, coded C71.7 in the Primary Site—labeled and with mainly type “9391/3; 9392/3; 9393/3” in the ICD‐O‐3 Hist/behav, malignant were screened.

### 2.2 Inclusion and Exclusion Criteria

**FIGURE 1:**
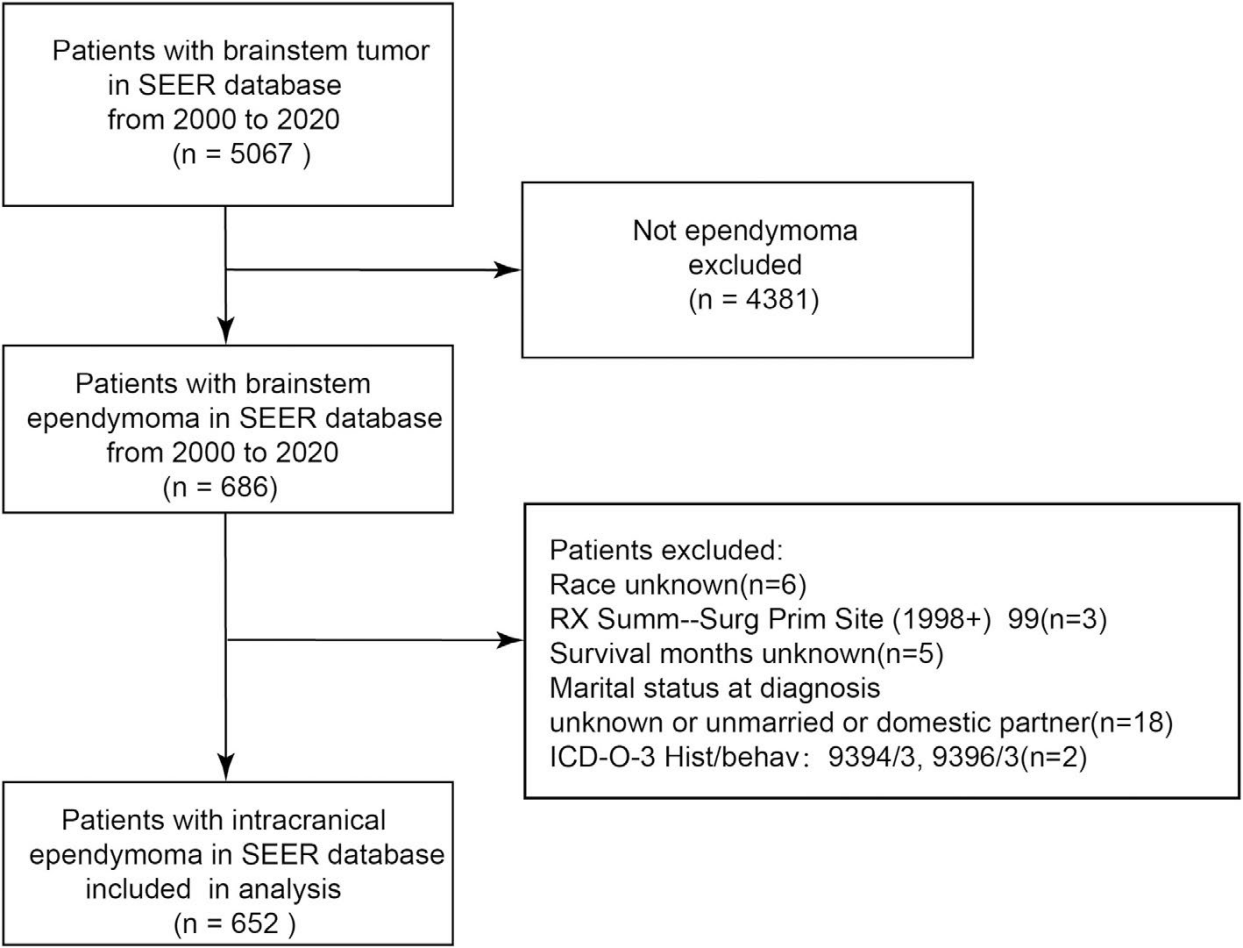
Flow chart of patient selection.

Figure 1 presents a flow chart showing the inclusion and exclusion criteria in the study.

The inclusion criteria as follows:

1. Diagnosed as ependymoma (2000–2020);
2. The primary site of ependymoma was in brainstem;
3. Demographic variables available: age, gender, race, and marital status;
4. Treatment regimens available: surgery resection, chemotherapy, and radiotherapy.

The exclusion criteria were following:

1. Race, RX Summ‐SurgPrim Site (1998+), Survival months unknown;
2. Marital status at diagnosis unknown or unmarried or domestic partner;

Finally, 652 patients were enrolled for the study of brainstem ependymoma.

### 2.3 Data Collection

Variables were extracted, such as patient age, gender, race, origin recode, marital status at diagnosis, tumor size, surgical status, radiation, ICD‐O‐3 Hist/behav, overall survival (OS) time, and status. Patients' races included white, black, and the others (American Indian/Alaska Native, Asian, or Pacific Islander) groups. DSW group was defined as patients whose marital status divorced, separated, and widowed according to previous literature [7]. We categorized the largest linear tumor size as≤or > 30 mm [8]. Tumor size was categorized as: 0–30 mm, 30+ mm, unknown. Surgical codes from RX Summ‐Surg Prim Site (1998+) were defined as no surgery and biopsy only for codes 00 and 20; partial resection for codes 21, 22, 40, and 90; and gross total resection (GTR) for codes 30 and 55, according to previous literature [1]. The ICD‐O‐3 Hist/behav codes of mainly pathological type in ependymoma included 9391/3 (Ependymoma, NOS), 9392/3 (Ependymoma, anaplastic), and 9393/3 (Papillary ependymoma, NOS).

### 2.4 Statistical Analysis

The software package R 4.2.0. were used for data analysis. Frequencies and percentages were used to summarize categorical variables. For continuous variables, the mean and standard deviation were reported if the data were normally distributed, while median and interquartile range were used for non‐normally distributed data. Nominal data comparisons employed the chi‐squared test or Fisher exact test. Differences between two groups were analyzed using the *t*‐test, Wilcoxon rank sum test, or chi‐squared test. A *p* value of less than 0.05 was considered statistical significance.

#### 2.4.1 Nomogram Construction and Validation

Patient age was categorized into two groups (< 50 and ≥ 50 years) on the basis of the optimal cutoff value determined using the survminer R package.

Univariate Cox regression analysis was conducted to identify potential prognostic variables. Patients were then randomly divided into a training set and a testing set in an 8:2 ratio using the createDataPartition function from the caret package. Variables (*p* < 0.15) of training set in univariate Cox regression were brought into multivariate Cox regression to identify the independent prognostic factors [9, 10].

Nomogram for predicting 1, 3, and 5 years overall survival probability was created based on the multivariate Cox regression model. The optimal threshold value for total points was derived using the “maxstat” method to categorize patients into high‐risk and low‐risk groups. Additionally, the receiver operating characteristic (ROC) curve and the area under the curve (AUC) was used to evaluate the model's discrimination ability. The uniformity between the nomogram and observed outcomes was assessed via Calibration curves.

#### 2.4.2 Kaplan–Meier Survival Analysis

We plotted the survival curves via Kaplan–Meier (K‐M) survival analysis. Surgical strategies were described in the RX Summ‐Surg Prim Site (1998+) and categorized into three classifications: no surgery and biopsy only, partial resection, GTR [1]. Regarding the surgical strategies, we analyzed the survival probability of patients in these three groups and between the GTR and no‐GTR group. We further compared the survival probabilities of patient with combined treatments (GTR, radiation, or chemotherapy).

Furthermore, we divided the patients into the following age groups: 0–19 years (the children group), 20–49 years (the adults' group), ≥ 50 years (the elderly group) [1] to analyze the overall survival of brainstem ependymoma patients in different age groups. Finally, we also compared the survival probability of patients with combined treatment (GTR, radiation, or chemotherapy) in different age groups to investigate the influence of the treatment regimens in different age patterns.

## 3 Results

### 3.1 Baseline Characteristics of the Study Population

A total of 652 patients with brainstem ependymomas, spanning all ages, were identified from 2000 to 2020. Median age of brainstem ependymomas patients at diagnosis was 26 years. Of these patients, 73.6% were under 50 years old, and 26.4% were 50 years or older. The gender distribution was 43.9% female (286 patients) and 56.1% male (366 patients). White patients constituted 84.2% of the cohort, and Spanish–Hispanic–Latino patients made up 25.6% of the total population. In terms of marital status, 58.3% were married, and 35.3% were single. Tumor size was ≤ 30 mm in 18.7% of patients and > 30 mm in 34.7% of patients. Gross total resection (GTR) was performed in 51.1% (333 patients), radiation therapy in 59.8% (390 patients), and chemotherapy in 15% (98 patients). Table 1 presents the characteristics of the study population.

**TABLE 1:** Distribution of demographics, tumor, and treatment characteristics of brainstem ependymoma patient from the SEER.

Variables	Overall	Training set	Validation set	*p*
Number	652	522	130	
Age, median(range)	26 (6–50)	25.00 (6.00, 50.00)	32.00 (5.00, 52.75)	0.709
Age, categorized				
< 50 years, *n* (%)	480 (73.6%)	387 (74.1%)	93 (71.5%)	0.624
≥ 50 years, *n* (%)	172 (26.4%)	135 (25.9%)	37 (28.5%)	
Gender				
Male, *n* (%)	366 (56.1%)	231 (44.3%)	55 (42.3%)	0.763
Female, *n* (%)	286 (43.9%)	291 (55.7%)	75 (57.7%)	
Race				
White, *n* (%)	549 (84.2%)	442 (84.7%)	107 (82.3%)	0.651
Black, *n* (%)	66 (10.1%)	50 (9.6%)	16 (12.3%)	
Others, *n* (%)	37 (5.7%)	30 (5.7%)	< 11 (5.4%)	
Spanish–Hispanic–Latino				
No, *n* (%)	485 (74.4%)	385 (73.8%)	100 (76.9%)	0.530
Yes, *n* (%)	167 (25.6%)	137 (26.2%)	30 (23.1%)	
Marital status at diagnosis				
Single, *n* (%)	380 (58.3%)	310 (59.4%)	70 (53.8%)	0.397
Married, *n* (%)	230 (35.3%)	181 (34.7%)	49 (37.7%)	
Divorced/widowed/separated, *n* (%)	42 (6.4%)	31 (5.9%)	11 (8.5%)	
Tumor size				
≤ 30 mm, *n* (%)	122 (18.7%)	100 (19.2)	22 (16.9)	0.673
> 30 mm, *n* (%)	226 (34.7%)	183 (35.1)	43 (33.1)	
Unknown, n (%)	304 (46.6%)	239 (45.8)	65 (50.0)	
Gross total resection				
No, *n* (%)	319 (48.9%)	251 (48.1%)	68 (52.3%)	0.445
Yes, *n* (%)	333 (51.1%)	271 (51.9%)	62 (47.7%)	
Radiation				
No, *n* (%)	262 (40.2%)	205 (39.3%)	57 (43.8%)	0.394
Yes, *n* (%)	390 (59.8%)	317 (60.7%)	73 (56.2%)	
Chemotherapy				
No/Unknown, *n* (%)	554 (85.0%)	444 (85.1%)	110 (84.6%)	1.000
Yes, *n* (%)	98 (15.0%)	78 (14.9%)	20 (15.4%)	
Histology				
9391/3: Ependymoma, NOS, *n* (%)	493 (75.6%)	386 (73.9%)	107 (82.3%)	0.130
9392/3: Ependymoma, anaplastic, *n* (%)	149 (22.9%)	127 (24.3%)	22 (16.9%)	
9393/3: Papillary ependymoma, NOS, *n* (%)	< 11 (1.5%)	< 11 (1.7%)	< 11 (0.8%)	

### 3.2 Feature Selection

**FIGURE 2:**
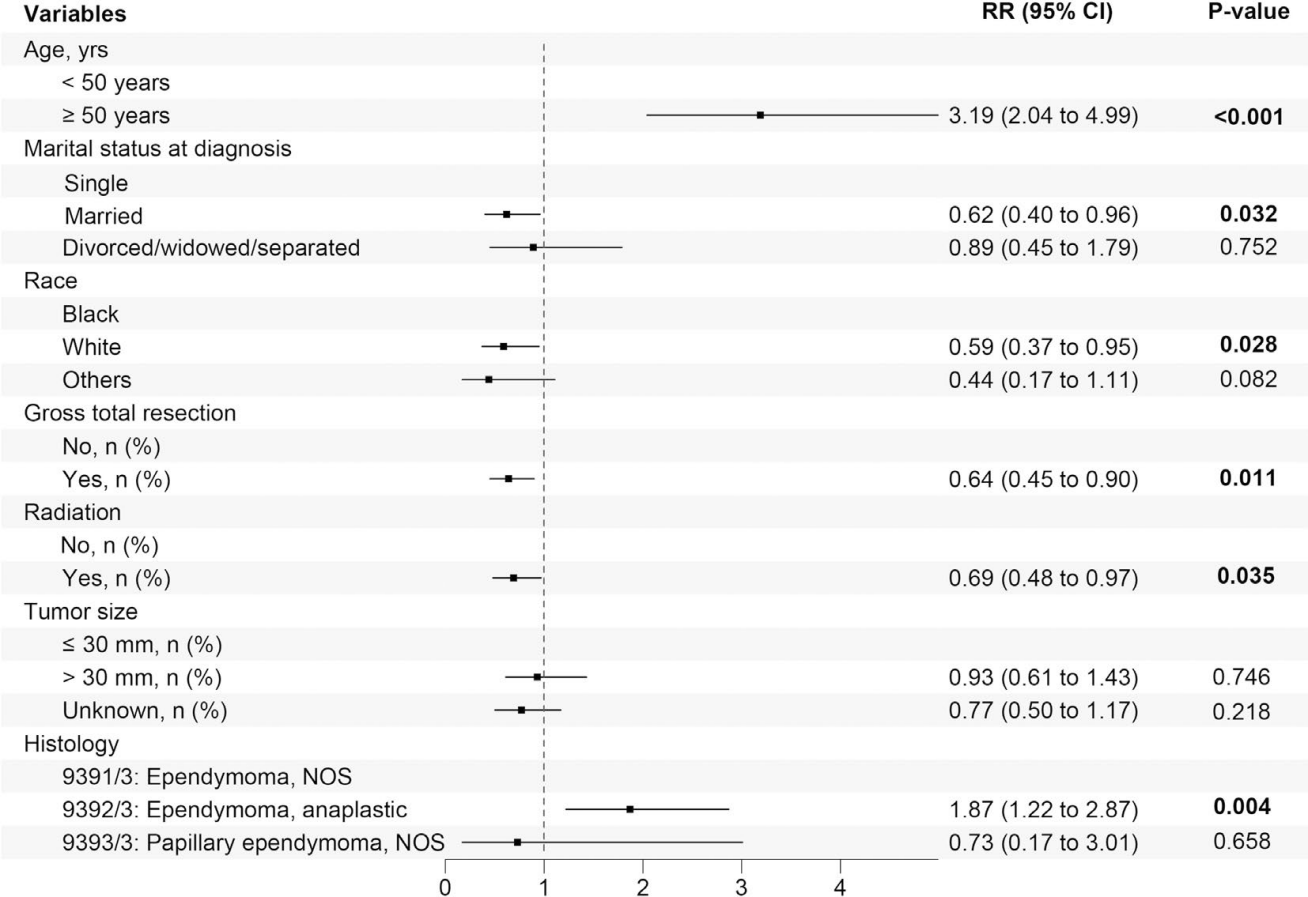
Forest plot for multivariate Cox regression analysis.

Patients were randomly divided into training and testing sets (8:2) using the caret package. The characteristics of the two groups are summarized in Table 1. Variables (*p* < 0.15) of training set in univariate Cox regression were brought into multivariate Cox regression to identify the independent prognostic factors (Table 2, Figure 2).

**TABLE 2:** Univariate Cox regression analysis of factors associated with overall survival of brainstem ependymoma.

Variables	OR (95% CI)	*p*
Age, years		
< 50 years	—	—
≥ 50 years	2.60 (1.87, 3.62)	< 0.001
Gender		
Female	—	—
Male	1.25 (0.90, 1.73)	0.192
Marital status at diagnosis		
Single	—	—
Married	1.04 (0.73, 1.47)	0.837
Divorced/widowed/separated	1.83 (1.00, 3.36)	0.051
Race		
Black	—	—
White	0.51 (0.32, 0.82)	0.005
Others	0.37 (0.15, 0.92)	0.032
Spanish–Hispanic–Latino		
No	—	—
Yes	1.24 (0.86, 1.78)	0.243
Gross total resection		
No, *n* (%)	—	—
Yes, *n* (%)	0.52 (0.37, 0.73)	< 0.001
Radiation		
No, *n* (%)	—	—
Yes, *n* (%)	0.58 (0.42, 0.80)	< 0.001
Chemotherapy		
No/Unknown, *n* (%)	—	—
Yes, *n* (%)	1.33 (0.86, 2.04)	0.201
Tumor size		
≤ 30 mm, *n* (%)	—	—
> 30 mm, *n* (%)	0.72 (0.47, 1.09)	0.118
Unknown, *n* (%)	0.73 (0.48, 1.11)	0.140
Histology		
9391/3: Ependymoma, NOS	—	—
9392/3: Ependymoma, anaplastic	1.32 (0.91, 1.91)	0.139
9393/3: Papillary ependymoma, NOS	0.58 (0.14, 2.34)	0.442

### 3.3 Nomogram Building and Validation

**FIGURE 3:**
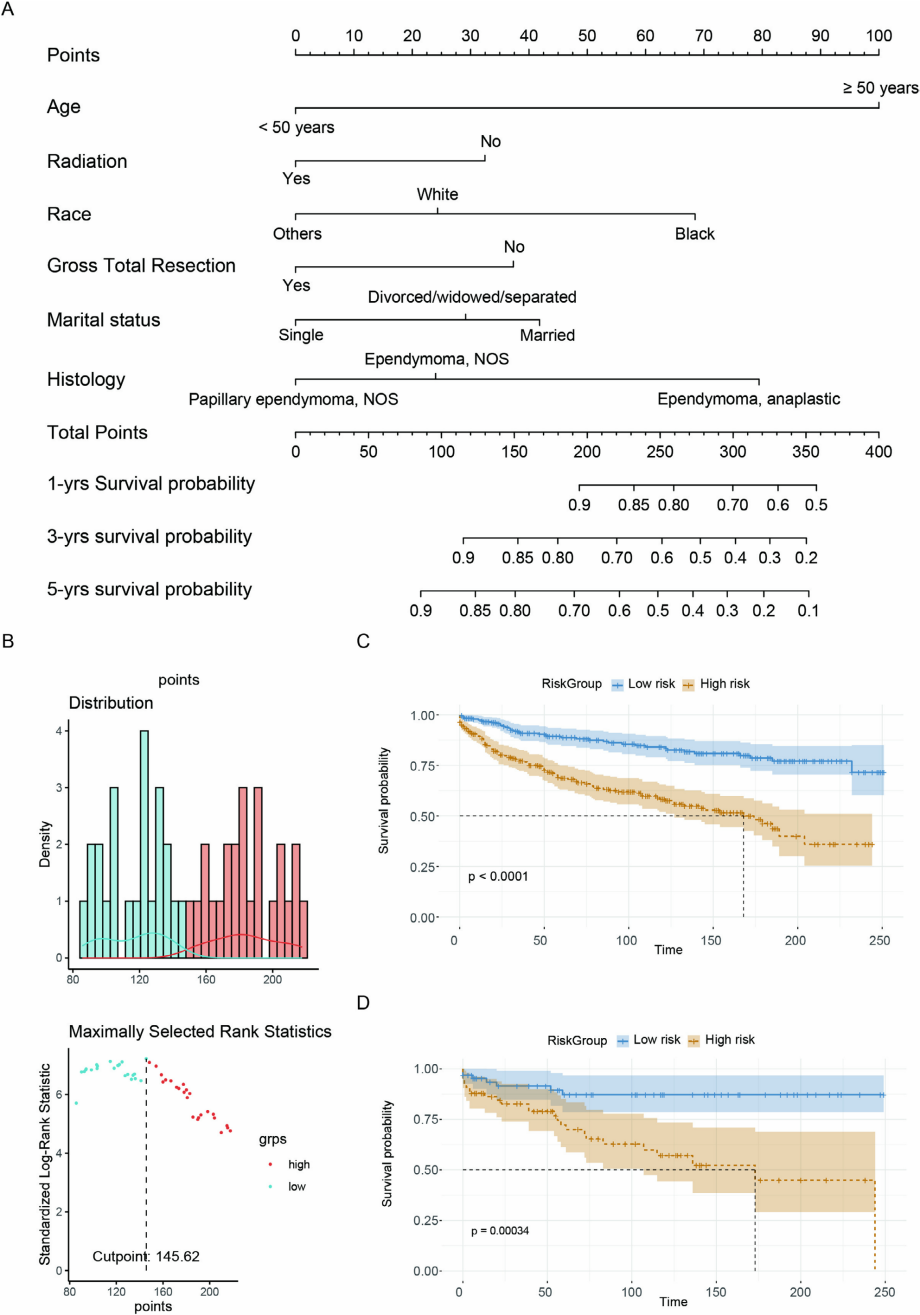
(A) Nomogram for brainstem ependymoma patients. (B) On the basis of the optimum cutoff value (145.62), patients were categorized into low‐risk and high‐risk groups. The Kaplan–Meier survival curve of these two groups: The training (C) and the testing (D) set.

A comprehensive model was developed utilizing six predictors identified through multivariate Cox regression analysis. To enhance the accessibility of the model, it was transformed into the form of a nomogram (Figure 3A), which are recognized as crucial tools in modern medical decision‐making, with a graphical representation of statistical prediction models. The optimal cutoff value for total points was derived using the “maxstat” method to categorize brainstem ependymoma patients into two groups: low‐risk and high‐risk (Figure 3B). K‐M survival curves showed that patients in the low‐risk group had a better prognosis compared with those in the high‐risk group (Figure 3C,D).

**FIGURE 4:**
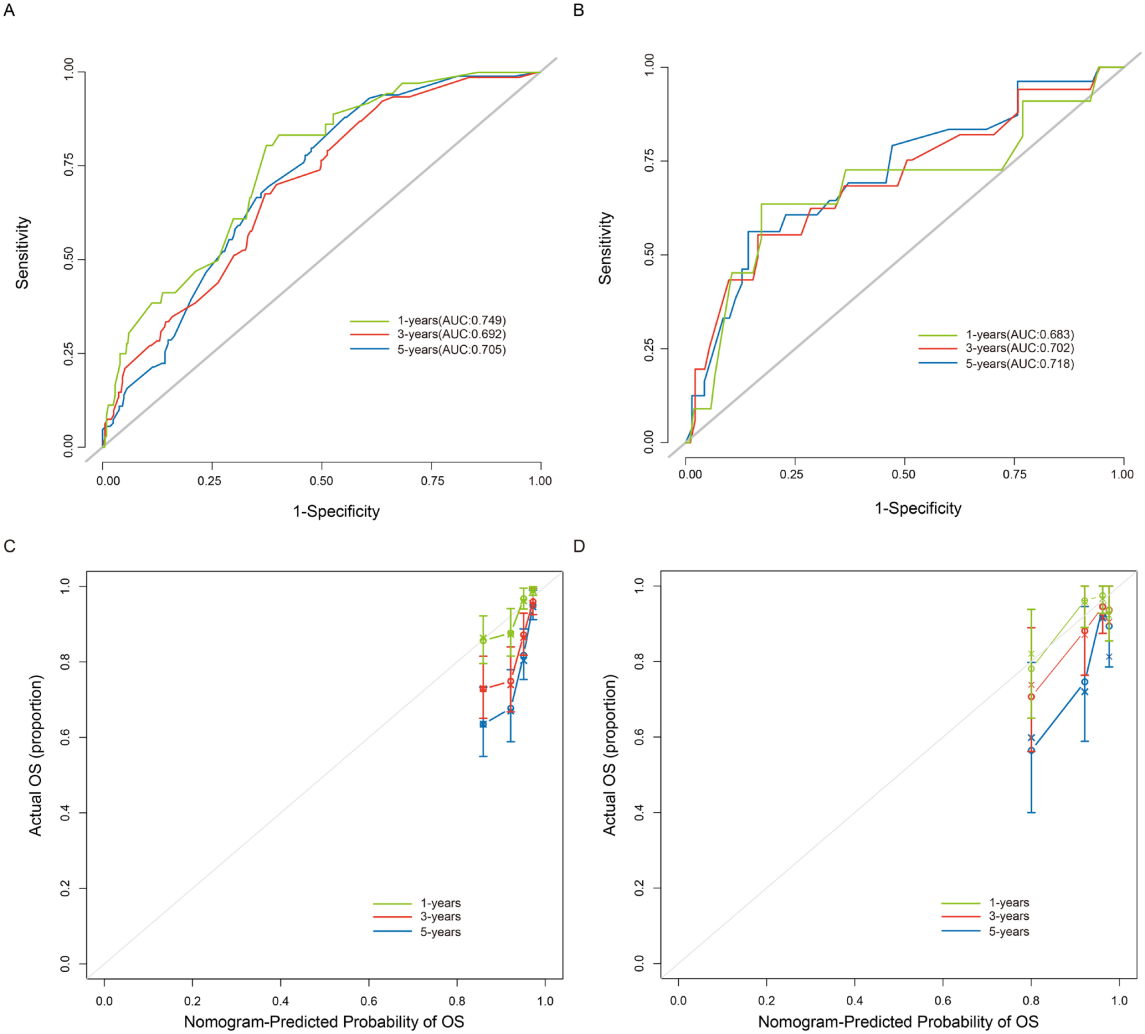
Receiver operating characteristic (ROC) curves of nomogram at 1, 3, and 5 years in the training set (A) and testing set (B). The calibration curve of the prognostic nomogram in the training set (C) and testing set (D).

Subsequently, ROC curve was conducted to assess the model's discrimination performance (Figure 4A,B). In addition, the calibration curve for overall survival probability also indicated a good uniformity between the nomogram and observed outcomes (Figure 4C,D).

### 3.4 Survival Analysis Based on Treatment Methods Stratification

**FIGURE 5:**
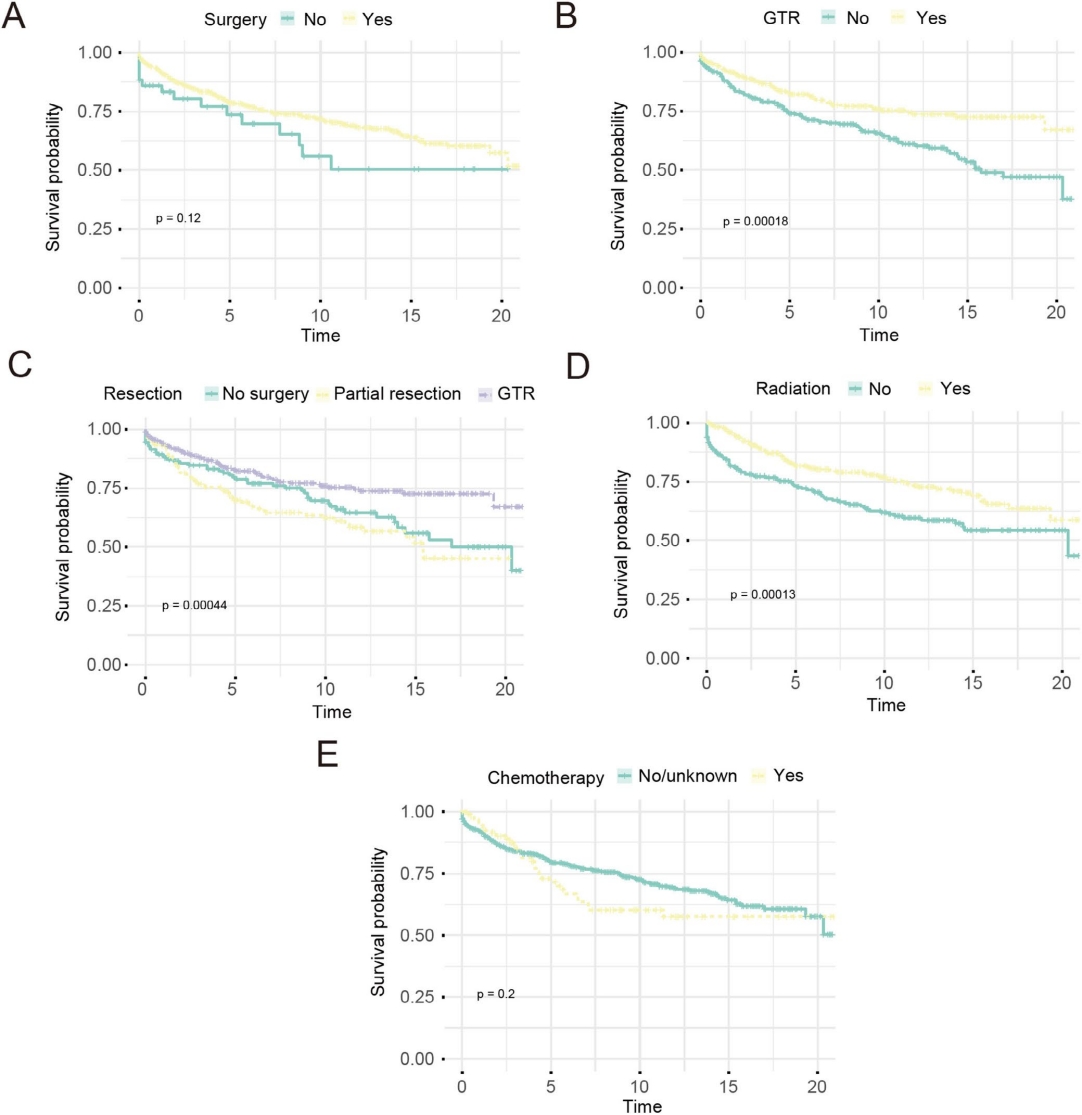
Kaplan–Meier survival curves based on treatment methods stratification. (A) surgery: *p* = 0.12; (B) gross total resection (GTR): *p* = 0.00018; (C) resection: No surgery vs. partial resection (*p* = 0.251), no surgery vs. GTR (*p* = 0.0004), partial resection vs. GTR (*p* = 0.022); (D) radiation: *p* = 0.00013; (E) chemotherapy: *p* = 0.2.

**FIGURE 6:**
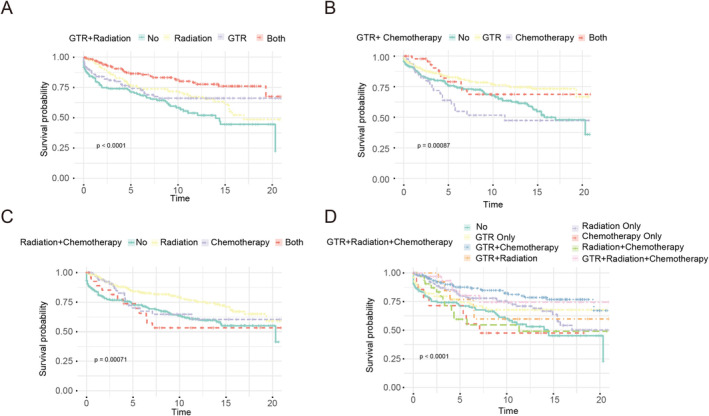
Kaplan–Meier survival curves based on combined treatment methods stratification. (A)GTR + radiation: GTR only vs. both (*p* = 0.014), radiation only vs. both (*p* = 0.0099), no vs. radiation only (*p* = 0.027); (B)GTR + chemotherapy: GTR only vs. no (*p* = 0.0037), GTR only vs. chemotherapy only (p = 0.0037), GTR only vs. both (*p* = 0.7456); (C) radiation+chemotherapy: Radiation only vs. no(*p* = 0.00073), chemotherapy only vs. no (*p* = 0.529), radiation only vs. both (*p* = 0.035); (D)GTR + radiation+chemotherapy: GTR + radiation vs. no (*p* < 0.0001), GTR + radiation vs. chemotherapy only (*p* = 0.007), GTR + chemotherapy only vs. chemotherapy only (*p* = 0.007).

Using Kaplan–Meier survival curves, we found that patients with GTR have improved overall survival than those without GTR (*p* = 0.00018) (Figure 5B), in addition to patients with no surgery and biopsy only or with partial resection (GTR vs. no: *p* = 0.0004, GTR vs. partial resection: *p* = 0.022) (Figure 5C). Patients who received radiation showed improved overall survival than patient without radiation (*p* = 0.00013) (Figure 5D). Patients who underwent GTR combined radiation therapy have improved overall survival than patient with or without GTR or radiation therapy alone (*p* < 0.0001) (Figure 6A).

### 3.5 Survival Analysis Based on Treatment Methods Stratification in Different Age Group

**FIGURE 7:**
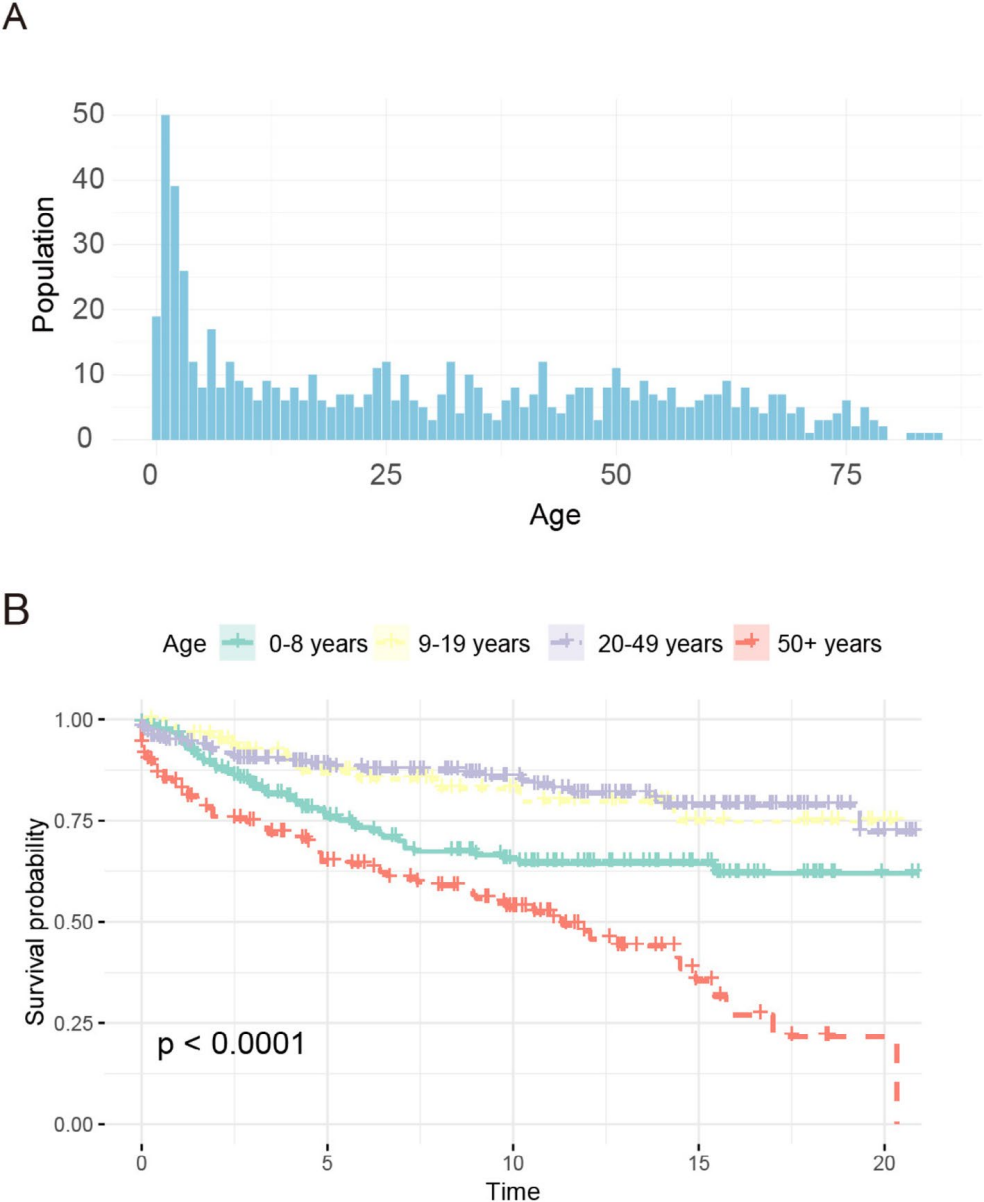
Age patterns for patients with brainstem ependymoma. (A) Age distribution of patients with brainstem ependymoma. (B) Kaplan–Meier survival curves of patients with brainstem ependymoma in different age groups.

The age distribution of patients with brainstem ependymoma is shown in Figure 7A. The population were divided into four different age groups (0–8 years[child group], 9–19 years [teenager group], 20–49 years [adult group], ≥ 50 years [elderly group]), to analyze the overall survival of patients in different age groups. Overall, we found that patients in the adult and teenager group had improved overall survival than patient in the child and the elderly group (adult vs. child: *p* = 0.002; adult vs. elderly: *p* < 0.0001; adult vs. teenager: *p* = 0.885; child vs. elderly: *p* = 0.0003; child vs. teenager: *p* = 0.035; elderly vs. teenager: *p* < 0.0001) (Figure 7B). in different age groups receiving combined treatment (GTR, radiation, or chemotherapy), finding that these different treatment methods had no significant effect on the overall survival probability of the elderly and teenager group, although GTR combined with radiation improved overall survival in the child and adult groups (Figure S1–S8).

## 4 Discussion

Brainstem ependymomas pose a unique challenge due to their complex and intricate functions. These tumors typically exhibit prolonged clinical progression and poor prognosis. Notable, the critical nature of brainstem function means that neurosurgeons view surgical resection as a significant challenge. Given the devastating natural history of brainstem ependymomas, it is important to investigate prognostic factors for survival in these patients. Efficient treatment strategies have also been explored.

### 4.1 Current Study

In recent years, an increasing number of scholars have utilized the SEER database for tumor research, leading to the development of various tumor prediction models. This trend may pave the way for new directions in tumor research in the future. To our knowledge, this is the largest study of patients with brainstem ependymoma, including 652 patients. This study is distinct from previous reports on this topic as it provides a nomogram of the survival of patients diagnosed with brainstem ependymomas, including over 20 years of data. Multivariate Cox regression analysis identified six prognostic predictors, with Black race and anaplastic ependymoma identified as risk factors. Furthermore, we found that patients aged < 50 years treated with combined, GTR and radiation exhibited better survival. Combined GTR and radiation therapy improved overall survival. There was no significant difference in OS on the elderly groups.

### 4.2 Analysis of Overall Survival

Surgery and radiotherapy are the primary treatment options for ependymomas in children [11, 12, 13]. Additionally, the benefits of postoperative radiotherapy have been demonstrated in terms of local control and survival rates in patients with intracranial ependymomas [14]. The primary treatment for adults with intracranial ependymoma is maximal surgical resection [15]. And adjuvant radiotherapy improves OS and progression‐free survival (PFS) [14, 16]. In this study, we found that GTR or radiotherapy accelerated the survival of patients with brainstem ependymoma. This is significant because there is currently no established consensus on differential management strategies based on age groups. Furthermore, research comparing treatment outcomes in different age groups with ependymomas is lacking. The present study found significantly longer survival in the younger population, with an age at diagnosis of 50 years. The poor prognosis of elderly patients with brainstem ependymomas may also be related to the shorter life expectancy in older individuals. This might need further studies to confirm.

Race has been identified as prognostic factors in various medical fields. In the present study, we confirmed ethnicity as a risk factor of ependymoma treatment outcomes. A previous study found that a higher extent of European genomic ancestry is associated with an increased risk of childhood ependymoma, whereas childhood ependymoma is less prevalent in African Americans and Native Americans [17]. Disparities in the incidence, mortality, and survival rates of ependymomas have been observed among various racial and ethnic groups. The present study indicated that black patients are at higher risk of succumbing to brainstem ependymoma. These racial differences in survival may indicate race as risk factors. Stenzel et al. also found that black pediatric patients with ependymoma had a higher risk of mortality among non‐Hispanic patients [18]. Rebecca L Achey al. found that African American populations had an increased risk of death compared with white populations [19]^(pp2000‐2016)^. However, it is possible that this gap in outcomes may be explained by socioeconomic risk factors, including household income, education level, and health care [18, 20]^(pp1975‐1999)^.

Significant variations in psychological and physical environments among married, single, separated, divorced, and widowed patients can lead to differing responses and treatment choices when faced with severe cancers. Previous research has examined the relationship between marital status and the survival of cancer patients, with the results of one study showing that married patients with glioma tend to have improved outcomes [7]. Research has examined the relationship between marital status and various cancer‐related factors, including cancer incidence, stage, treatment options, and prognosis. Notably, a recent study indicated that unmarried adult patients with ependymomas had a higher risk of poorer outcome [21].

The extent of resection is a crucial predictor of outcomes for ependymomas [16]. However, the location of the tumor, such as in the posterior fossa affecting the brainstem, can limit surgery due to the involvement of the lower cranial nerves and brainstem, making complete resection difficult [22]. The five‐year OS rate was 70% in patients who underwent GTR. In contrast, OS rates were lower when resection was incomplete [11]. In our study, we observed longer survival in patients who underwent GTR. Additionally, adjuvant radiation therapy provided further tumor control, contributing to improved outcomes. This is consistent with previous studies [23, 24].

There is a consensus among medical professionals to incorporate postoperative radiotherapy into the standard care for adult patients with anaplastic ependymoma classified as Grade II or III by the World Health Organization (WHO) classification, particularly following an incomplete resection [11]. Furthermore, there is limited evidence to support the effectiveness of chemotherapy and targeted agents in the treatment of adult ependymomas. Patients with anaplastic ependymoma exhibited a 2‐fold increased risk of mortality compared to those with ependymoma, NOS [19].

### 4.3 Analysis of Combined Treatments

In this study, GTR improved overall survival compared to patients without GTR, while patients with GTR showed improved overall survival compared to patients with no surgery and biopsy only or with partial resection. Patients who underwent GTR combined with radiation therapy had improved overall survival than patients without or with GTR or radiation therapy only.

GTR has previously been associated with a reduced risk of tumor recurrence and residual tumor bleeding, while stereotactic radiotherapy has been significantly linked to better outcomes [12]. Meanwhile, the toxicity of radiotherapy makes the brain at risk during radiation therapy, especially in younger children [11]. We found that patients in the adult and teenager group had improved overall survival compared with patients in the children and elderly groups. Studies on chemotherapy for ependymomas are lacking. Chemotherapy with temozolomide prevents tumor progression in individual cases [23]. In the present study, we found that it was difficult for chemotherapy to accelerate survival compared with other therapies. Different treatment methods showed no significant difference in the overall survival probability of the elderly group, and GTR combined with radiation improved overall survival in children and adults. However, further evidence is required in future studies.

## 5 Limitations

Our analysis has several limitations that must be considered. First, some of the important information investigated, such as tumor location (midbrain, pons, and medulla), types of tumor growth, molecular pathology, specific treatment details, and tumor progression, had limited availability. Specific locations and molecular characteristics are crucial factors influencing disease progression, and molecular pathological assessments are widely utilized in clinical practice. Furthermore, the five‐year survival rate is associated with tumor location, with midbrain lesions demonstrating the most favorable outcome and pontine lesions showing the poorest prognosis [25]. Second, we conducted only internal verification of the data, with the aim of obtaining external validation in real‐world settings in the future. Third, to enhance the model's generalizability, we excluded the pathological types 9394 (*n* = 1) and 9396 (*n* = 1) from this study and focused only on the three main pathological types. We hope to include more data on these two rare pathological types in the future research. Finally, the ventrolateral exophytic brainstem ependymoma category might include fourth ventricular ependymoma; however, this distinction is not specified within the SEER database. We hope that further details can be obtained from the SEER database in the future.

## 6 Conclusion

Overall, the present study identified individuals who are Black or anaplastic ependymomas are negative factors for brainstem ependymomas, with an increased risk of mortality. Patients aged < 50 years with GTR and radiation always showed better survival.

## Author Contributions

**Xiaoyu Ji:** conceptualization (equal), investigation (equal), methodology (equal), supervision (equal), validation (equal), visualization (equal), writing – original draft (equal), writing – review and editing (equal). **Siyuan Yang:** conceptualization (equal), data curation (equal), formal analysis (equal), investigation (equal), methodology (equal), software (equal), writing – original draft (equal), writing – review and editing (equal). **Dejing Cheng:** data curation (equal), software (equal), writing – original draft (equal). **Wenbo Zhao:** data curation (equal), software (equal), supervision (equal), validation (equal). **Xuebo Sun:** conceptualization (equal), investigation (equal), methodology (equal), supervision (equal), validation (equal), visualization (equal), writing – review and editing (equal). **Fang Su:** conceptualization (equal), supervision (equal), validation (equal), visualization (equal), writing – review and editing (equal).

## Ethics Statement

This study is a retrospective analysis involving data from public databases. Therefore, ethical approval was not necessary.

## Conflicts of Interest

The authors declare no conflicts of interest.

## Supporting information


**Figure S1.** Kaplan–Meier survival curves based on treatment methods stratification in 0–8 years old groups.
**Figure S2.** Kaplan–Meier survival curves based on combined treatment methods stratification in 0–8 years old groups.
**Figure S3.** Kaplan–Meier survival curves based on treatment methods stratification in 9–19 years old groups.
**Figure S4.** Kaplan–Meier survival curves based on combined treatment methods stratification in 9–19 years old groups.
**Figure S5.** Kaplan–Meier survival curves based on treatment methods stratification in 20–49 years old groups.
**Figure S6.** Kaplan–Meier survival curves based on combined treatment methods stratification in 20–49 years old groups.
**Figure S7.** Kaplan–Meier survival curves based on treatment methods stratification in 50+ years old groups.
**Figure S8.** Kaplan–Meier survival curves based on combined treatment methods stratification in 50+ years old groups.

## Data Availability

The dataset from the SEER database that was generated and/or analyzed during the current study is available in the SEER dataset repository (https://seer.cancer.gov/). The datasets generated during and/or analyzed during the current study are available from the corresponding author upon reasonable request.
