# Supplementary material for: Prognostic Factors and Nomogram for Malignant Brainstem Ependymoma: A Population‐Based Retrospective Surveillance, Epidemiology, and End Results Database Analysis
Source: Cancer Med. 2025 Jan 17;14(2):e70564. doi: 10.1002/cam4.70564 (PMC11756553; doi:10.1002/cam4.70564)

**Supplementary material**

**Figure S1 Kaplan-Meier survival curves based on treatment methods stratification in 0-8 years old groups.**


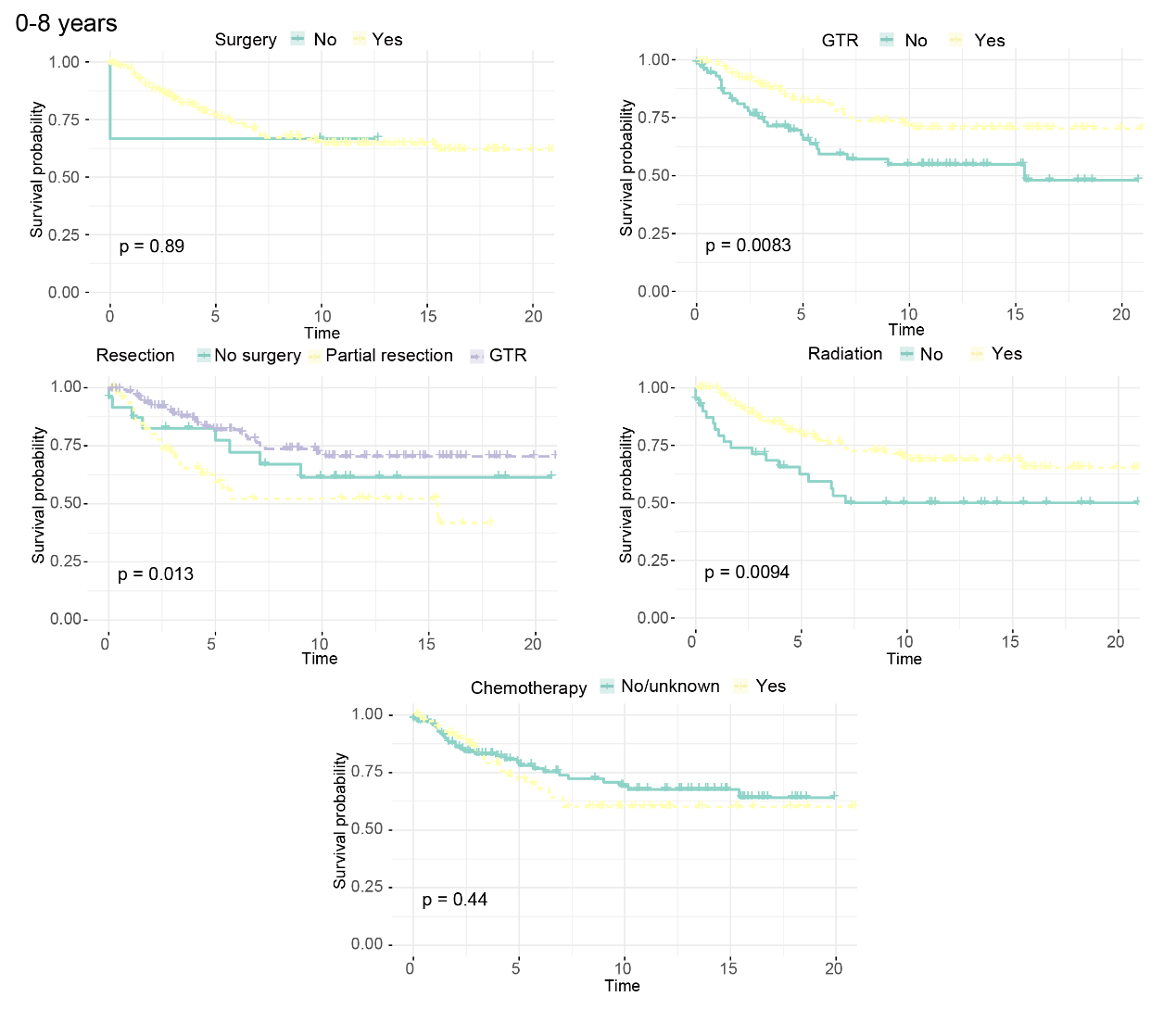


**Figure S2 Kaplan-Meier survival curves based on combined treatment methods stratification in 0-8 years old groups.**


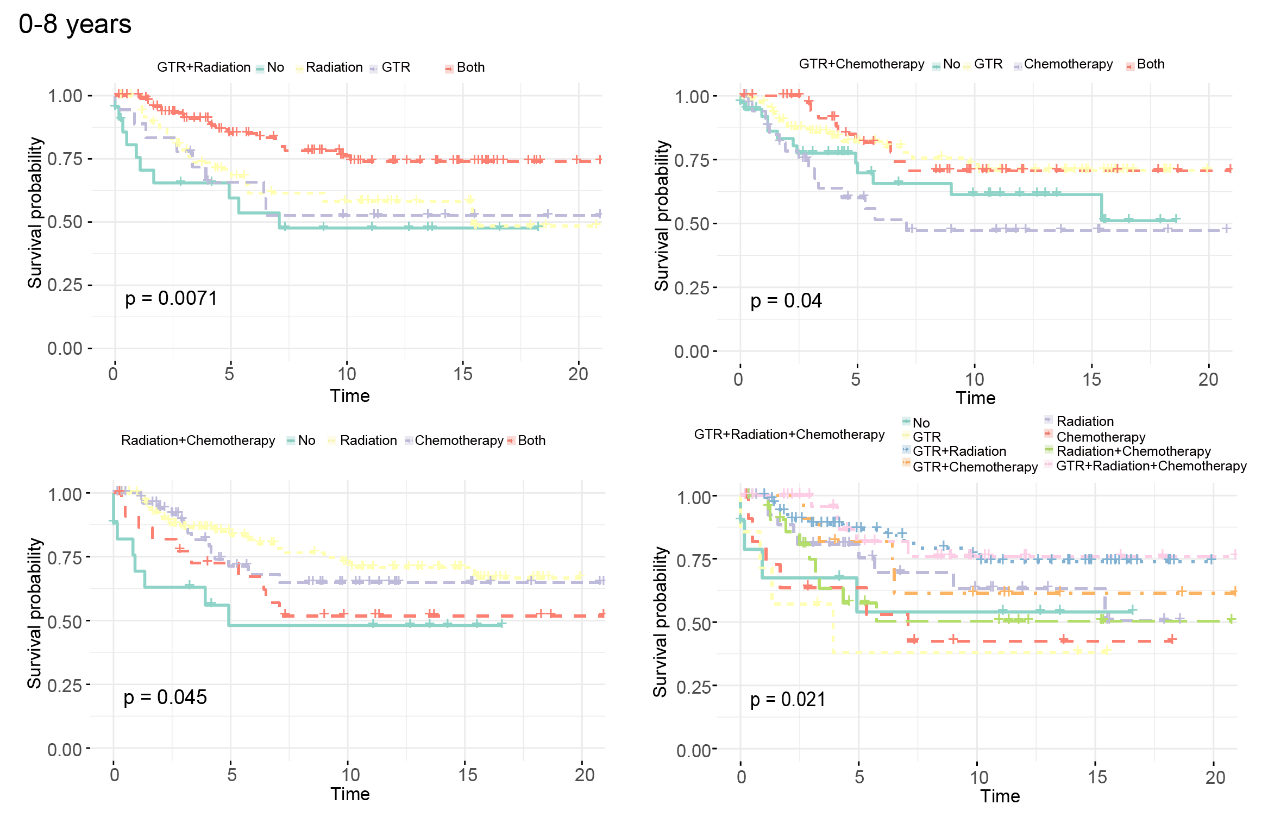


**Figure S3 Kaplan-Meier survival curves based on treatment methods stratification in 9-19 years old groups.**


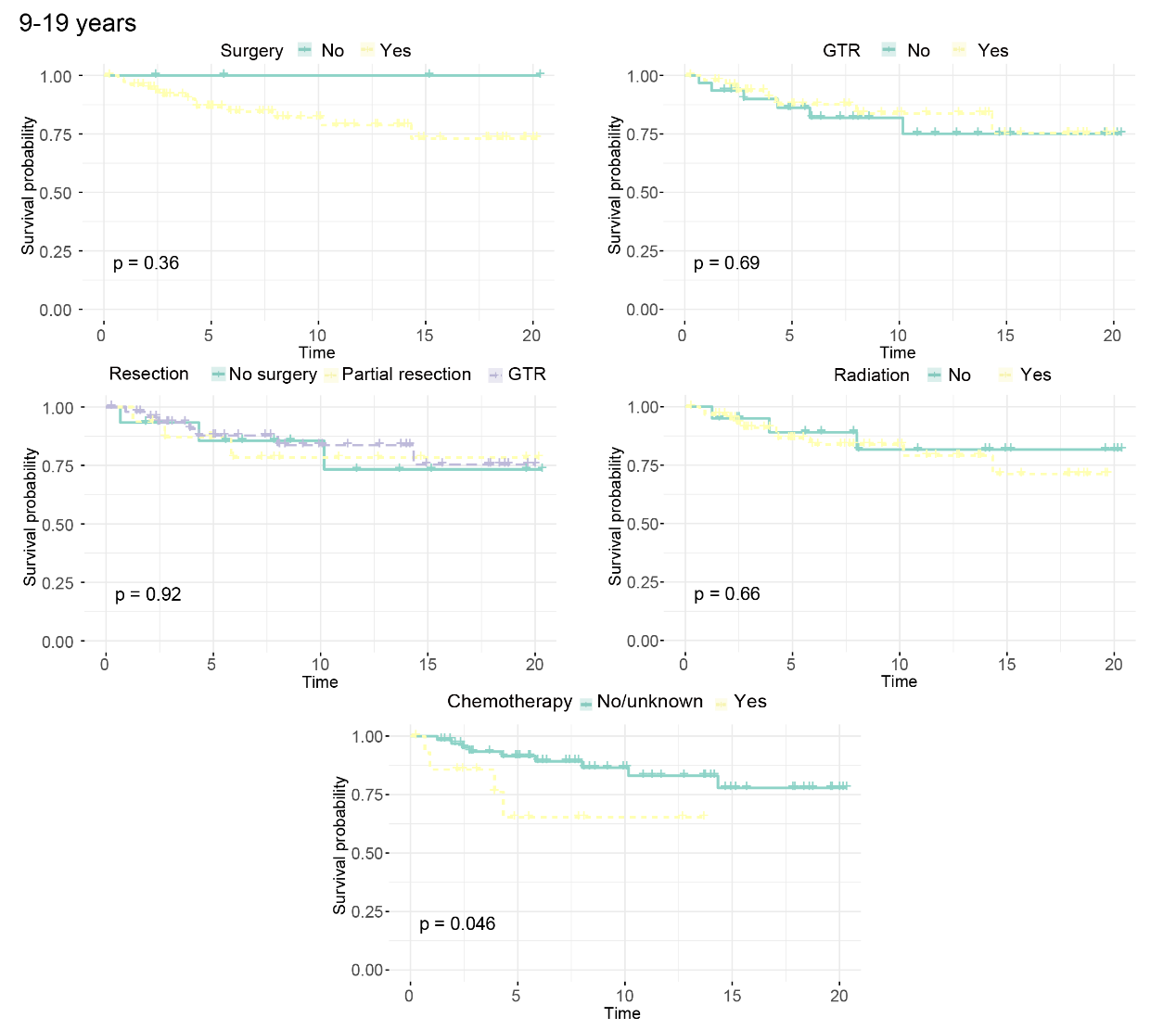


**Figure S4 Kaplan-Meier survival curves based on combined treatment methods stratification in 9-19 years old groups.**


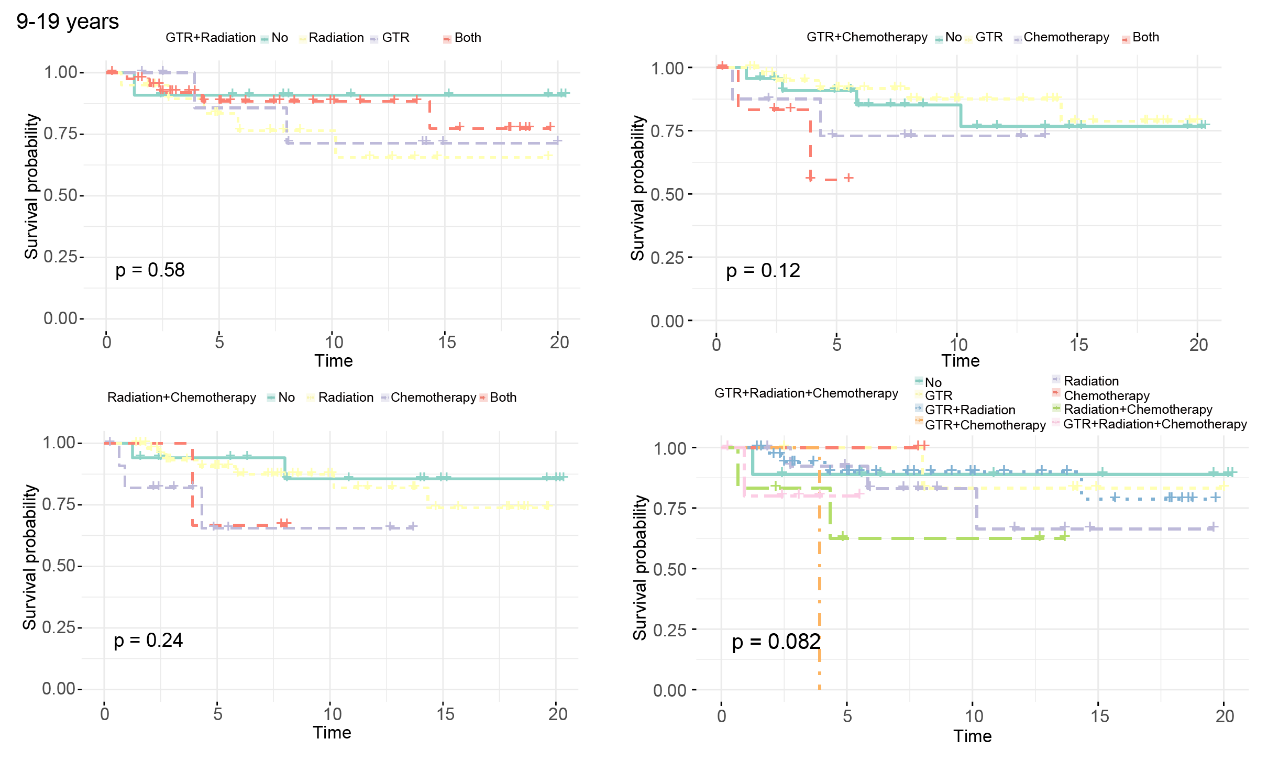


**Figure S5 Kaplan-Meier survival curves based on treatment methods stratification in 20-49 years old groups.**


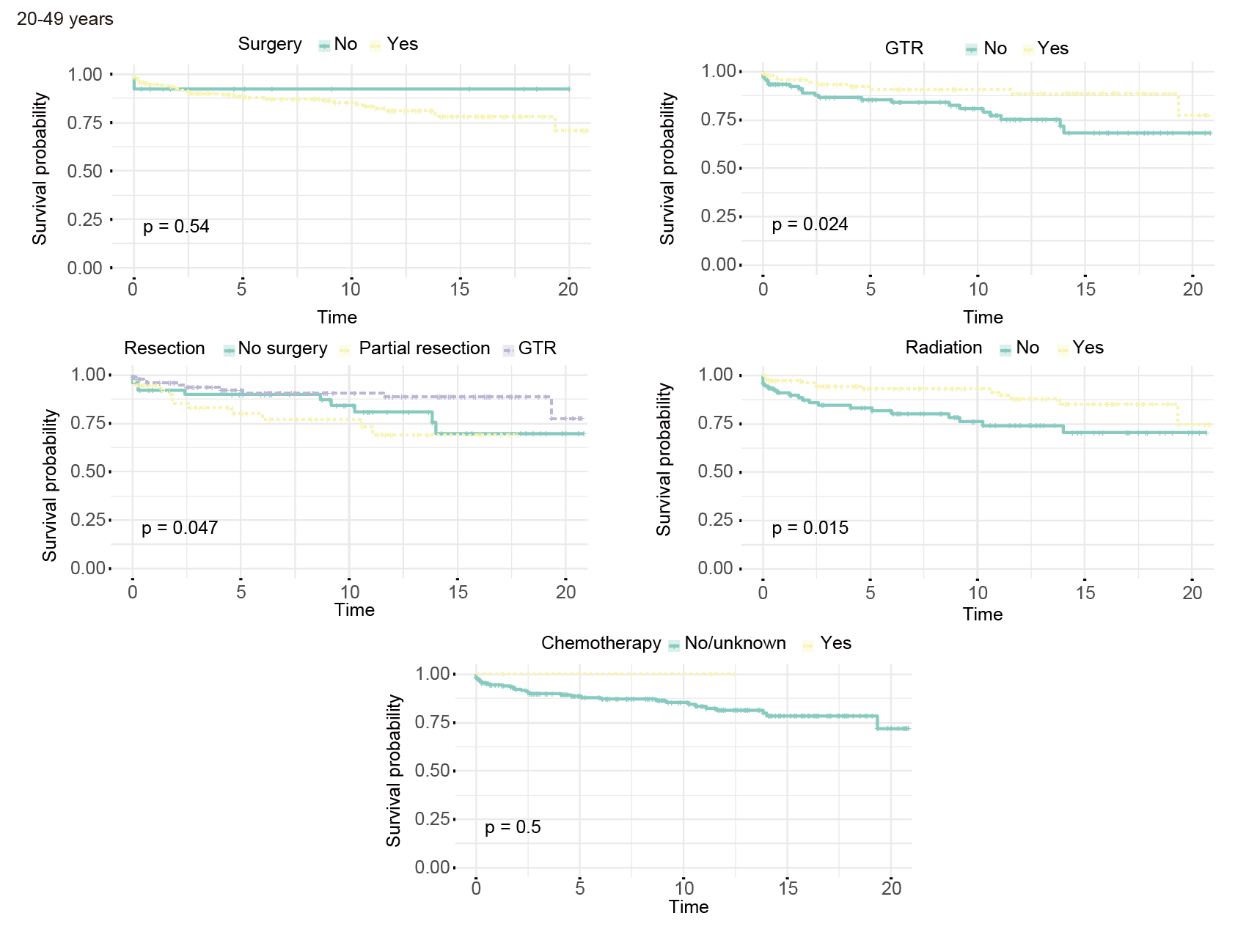


**Figure S6 Kaplan-Meier survival curves based on combined treatment methods stratification in 20-49 years old groups.**


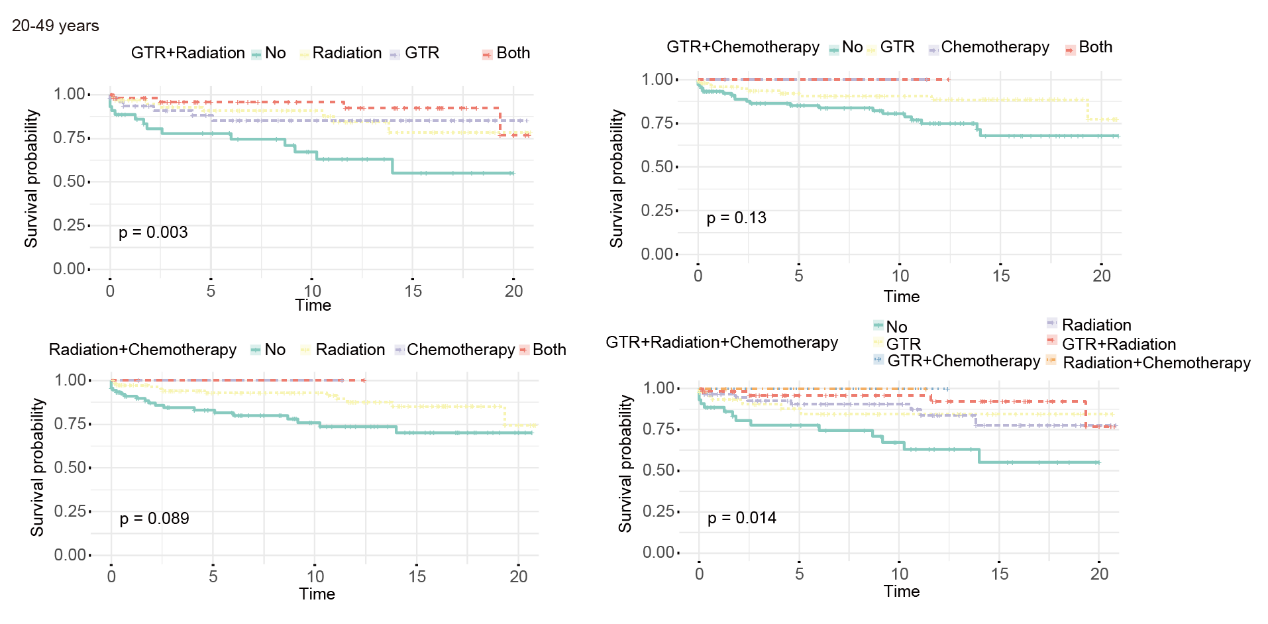


**Figure S7 Kaplan-Meier survival curves based on treatment methods stratification in 50+ years old groups.**


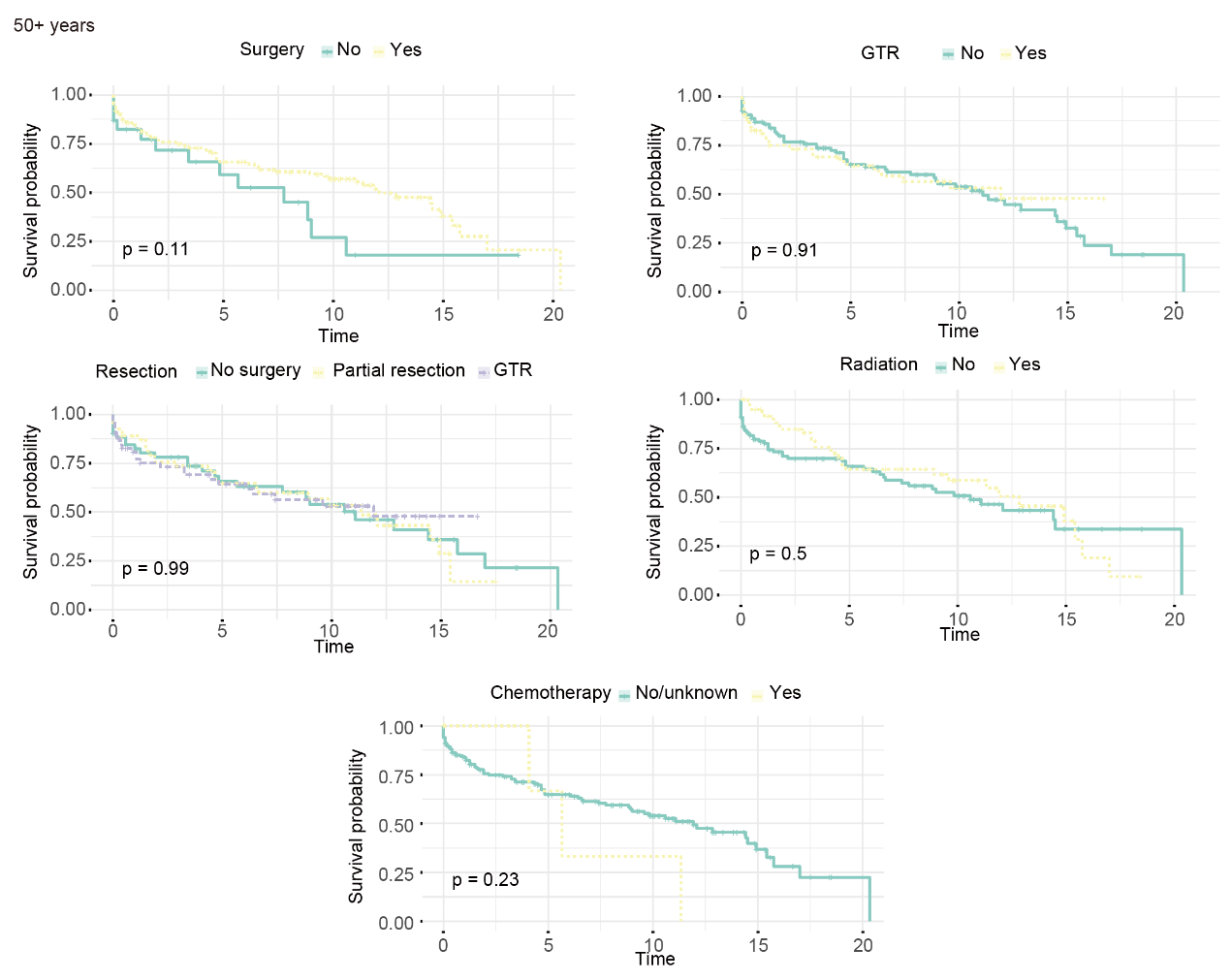


**Figure S8 Kaplan-Meier survival curves based on combined treatment methods stratification in 50+ years old groups.**


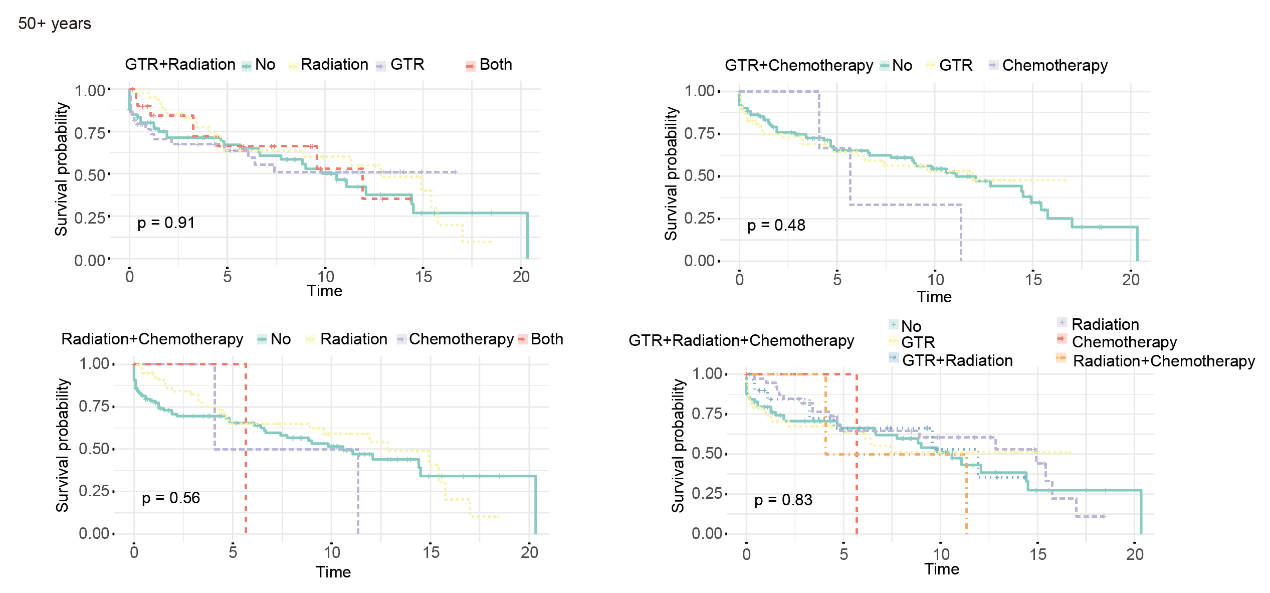

Supplement: Supplementary file 1 — Figure S1. Kaplan–Meier survival curves based on treatment methods stratification in 0–8 years old groups. Figure S2. Kaplan–Meier survival curves based on combined treatment methods stratification in 0–8 years old groups. Figure S3. Kaplan–Meier survival curves based on treatment methods stratification in 9–19 years old groups. Figure S4. Kaplan–Meier survival curves based on combined treatment methods stratification in 9–19 years old groups. Figure S5. Kaplan–Meier survival curves based on treatment methods stratification in 20–49 years old groups. Figure S6. Kaplan–Meier survival curves based on combined treatment methods stratification in 20–49 years old groups. Figure S7. Kaplan–Meier survival curves based on treatment methods stratification in 50+ years old groups. Figure S8. Kaplan–Meier survival curves based on combined treatment methods stratification in 50+ years old groups. [file CAM4-14-e70564-s001.docx]
